# Disaster eHealth: Scoping Review

**DOI:** 10.2196/18310

**Published:** 2020-10-28

**Authors:** Samaneh Madanian, Tony Norris, Dave Parry

**Affiliations:** 1 Auckland University of Technology (AUT) Auckland New Zealand

**Keywords:** medical informatics, disaster planning, disaster medicine, medical informatics applications, disasters

## Abstract

**Background:**

Although both disaster management and disaster medicine have been used for decades, their efficiency and effectiveness have been far from perfect. One reason could be the lack of systematic utilization of modern technologies, such as eHealth, in their operations. To address this issue, researchers’ efforts have led to the emergence of the disaster eHealth (DEH) field. DEH’s main objective is to systematically integrate eHealth technologies for health care purposes within the disaster management cycle (DMC).

**Objective:**

This study aims to identify, map, and define the scope of DEH as a new area of research at the intersection of disaster management, emergency medicine, and eHealth.

**Methods:**

An extensive scoping review using published materials was carried out in the areas of disaster management, disaster medicine, and eHealth to identify the scope of DEH. This review procedure was iterative and conducted in multiple scientific databases in 2 rounds, one using controlled indexed terms and the other using similar uncontrolled terms. In both rounds, the publications ranged from 1990 to 2016, and all the appropriate research studies discovered were considered, regardless of their research design, methodology, and quality. Information extracted from both rounds was thematically analyzed to define the DEH scope, and the results were evaluated by the field experts through a Delphi method.

**Results:**

In both rounds of the research, searching for eHealth applications within DMC yielded 404 relevant studies that showed eHealth applications in different disaster types and disaster phases. These applications varied with respect to the eHealth technology types, functions, services, and stakeholders. The results led to the identification of the scope of DEH, including eHealth technologies and their applications, services, and future developments that are applicable to disasters as well as to related stakeholders. Reference to the elements of the DEH scope indicates what, when, and how current eHealth technologies can be used in the DMC.

**Conclusions:**

Comprehensive data gathering from multiple databases offered a grounded method to define the DEH scope. This scope comprises concepts related to DEH and the boundaries that define it. The scope identifies the eHealth technologies relevant to DEH and the functions and services that can be provided by these technologies. In addition, the scope tells us which groups can use the provided services and functions and in which disaster types or phases. DEH approaches could potentially improve the response to health care demands before, during, and after disasters. DEH takes advantage of eHealth technologies to facilitate DMC tasks and activities, enhance their efficiency and effectiveness, and enhance health care delivery and provide more quality health care services to the wider population regardless of their geographical location or even disaster types and phases.

## Introduction

Disasters are destructive events that threaten public health and the environment and disrupt and/or impede normal operations. They also impose considerable pressure on health care systems. The source of disasters can be natural or the result of human actions (eg, fires and terrorist attacks) [1]. Disease epidemics can also be seen as disasters, albeit over a longer time scale rather than a point event, killing thousands of people or making them homeless, destroying health infrastructure, and disrupting public and commercial services. Data from the Centre for Research on the Epidemiology of Disasters (CRED) [2] show that the severity and complexity (damage to life and property) of disasters have grown exponentially over recent decades. In 2018, 331 natural disasters were identified; these caused 14,854 deaths, affected a further 81,143,283 and cost US $130,655,327,000.

Disaster management and disaster medicine are complementary disciplines that can significantly reduce the harmful effects of disasters. Disaster management conveniently encompasses 4 phases: mitigation, preparedness, response, and recovery [3]. Addressing issues arising in these phases requires good collaboration among different governmental and nongovernmental organizations and disaster medicine groups. However, both disaster management and disaster medicine operations are frequently far from perfect and have a long list of failures [4]. In some cases, disaster medicine groups have poor communication with disaster management organizations; in this regard [5], claims *emergency management and the health sectors are natural allies that have, seemingly, only recently begun to recognize each other*. According to the reviewed literature, some major contributions to this issue are as follows:

1. Disaster management and disaster medicine have different roots, development, and priorities [6]. Therefore, coordination and communication between them in disasters are often missing, which leads to delayed, substandard, improper, or sometimes no care.
2. Although both areas emerged to work side by side, they sometimes fail to share their tools and personnel and have not collaborated smoothly in preparing for and responding to mass emergencies.
3. Neither disaster medicine nor disaster management routinely uses information or modern eHealth technologies [6].

Therefore, there is a pressing need for efficient disaster management and emergency medicine to mitigate human pain and suffering and the overall impact of disasters [1]. These issues are the reasons that renewed interest in this field has appeared and necessitates more research.

**Figure 1:**
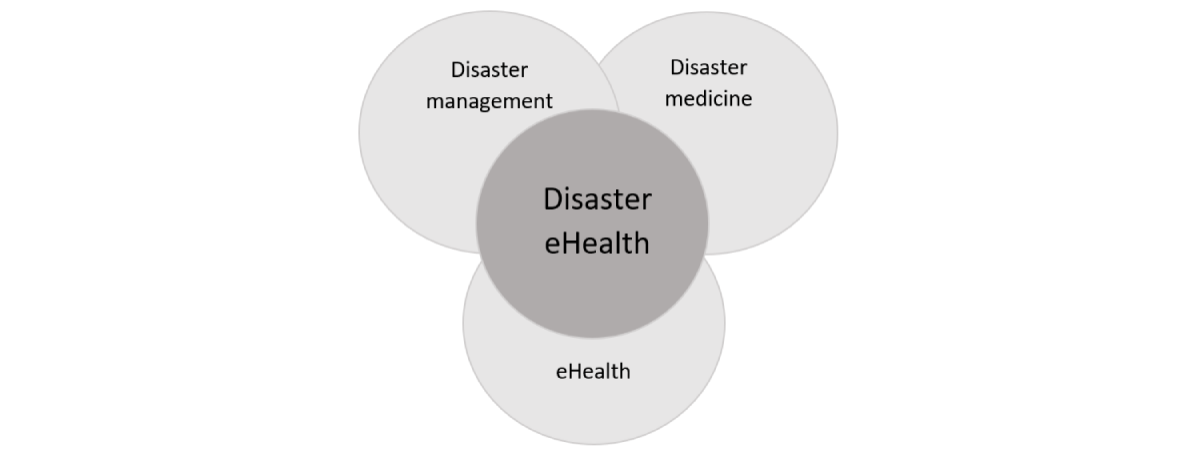
Disaster eHealth and its components.

Despite the growth of information technology capacities and services (eg, new communication technologies, ubiquitous computing, the internet, and advanced smart devices [7]) in mainstream health care, their applications in disaster management and disaster medicine fields are currently limited. However, they offer great potential for improving efficiency and effectiveness in disaster situations, especially when eHealth technologies are integrated and applied systematically throughout the disaster management cycle (DMC) [7] to improve disaster health planning before, response, during, and recovery after disasters. To achieve this goal, the domain of disaster eHealth (DEH) has been proposed in the study by Norris et al on 2015 [6] at the intersection of disaster management, disaster medicine, and eHealth fields (Figure 1). The definitions of these terms are provided in Textbox 1.

DEH is an emerging field that was introduced earlier in the study by Norris et al [6]. In that research, besides the definition of DEH, its vision was defined. However, as DEH has not yet been generally acknowledged, profiling and defining the field and its scope and assessing the role of eHealth technologies in each phase of disasters are important. Therefore, the aim of this study is to define the scope of DEH rather than providing detailed analysis and reviewing the literature related to the field of DEH.

DEH can be seen as a model telling us what, when, and how current eHealth technologies can be used in the DMC. These technologies include not only those used in established eHealth practices but also those recently made available by the rapid development in mobile and sensor technologies.

**Disaster eHealth and the definition of its components.**

- Disaster eHealth: the application of information and eHealth technologies in a disaster situation to restore and maintain the health of individuals to their predisaster levels
- Disaster management: the coordination and integration of all activities necessary to build, sustain, and improve the capabilities to prepare for, respond to, recover from, or mitigate against threatened or actual disasters or emergencies, regardless of cause [8]
- Disaster medicine: a system of study and medical practice associated primarily with the disciplines of emergency medicine and public health [9]
- eHealth: the cost-effective and secure use of information and communications technology in support of health and health-related fields, including health care services, health surveillance, health literature, and health education, knowledge, and research [10]

## Methods

### Study Design

**Figure 2:**
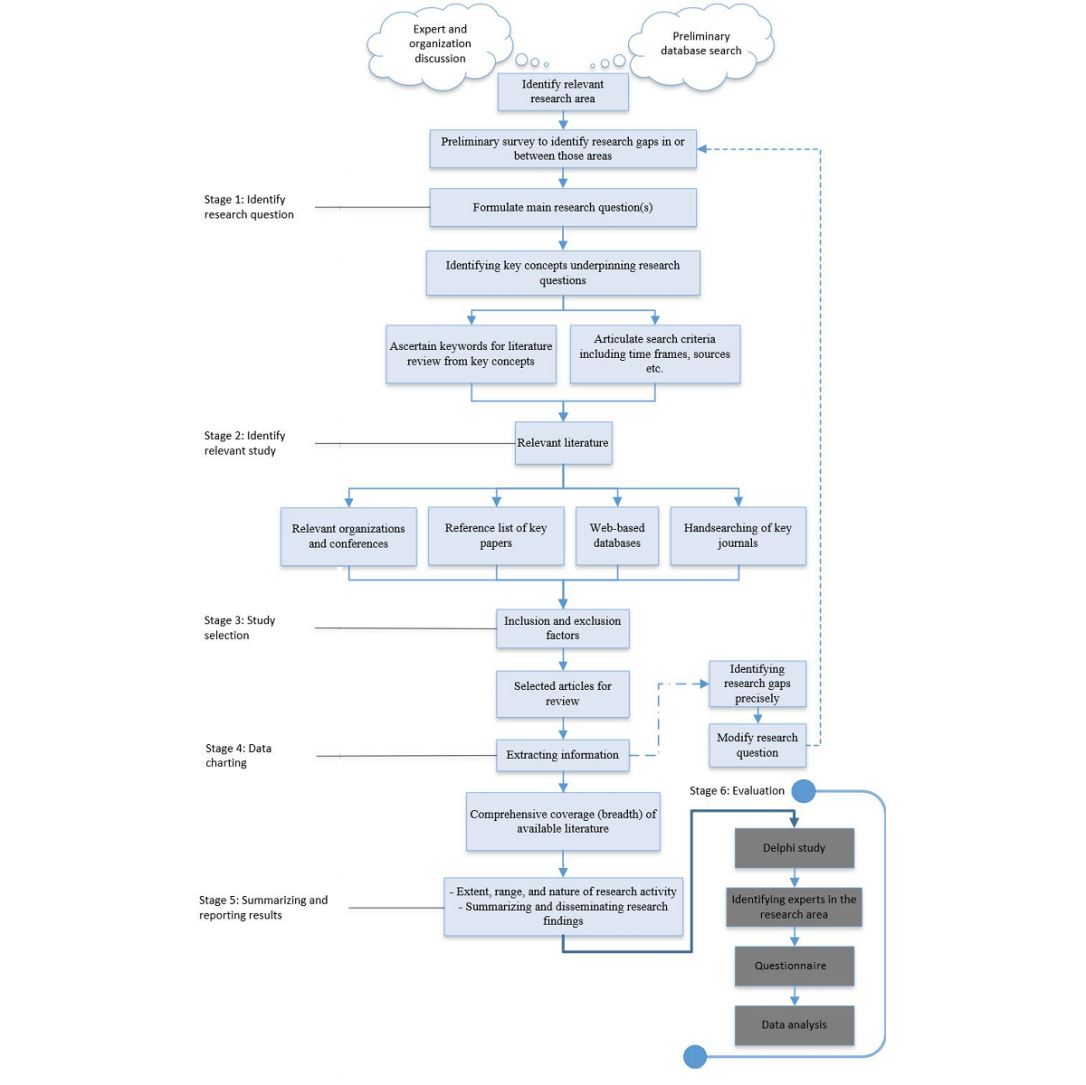
The scoping study framework for disaster eHealth.

To undertake this research, a rigorous scoping study was conducted based on the framework of Arksey and O’Malley [11]. Our adapted framework included an additional stage to evaluate the research results, as suggested by Levac et al [12]. The adapted framework is presented in Figure 2.

This research is limited to publications from 1990 to 2016. This scoping study was undertaken in 2 complementary rounds: uncontrolled and controlled search. The uncontrolled search was commenced in multiple databases using free-text terms rather than indexed terms. This approach uses a search engine to identify documents of interest based on terms occurring in the papers’ titles, abstracts, or main bodies. This allowed us to extensively and fully explore the area and extract a broad range of articles to define the scope of DEH. However, to improve the accuracy of free-text search and to decrease the potential searching bias or missing data in the search, a controlled search was employed [13]. In this method, keywords were selected from an existing list of index terms on which articles are indexed. The method is used in particular databases, such as MeSH in PubMed and Controlled Indexing in IEEE Xplore. A sample list of searched terms and queries in both research procedures is provided in Table 1.

**Table 1:** A sample of search terms and queries.

Searching terms	Uncontrolled search	Controlled search
Search terms	disaster management disaster disaster medicine ehealth e*health	disaster medical informatics disaster medicine emergency management
Search queries	(ehealth OR e-health OR e*health) AND disaster (ehealth OR e-health OR e*health) AND “disaster medicine”	(“Disasters”[Mesh]) AND “Medical Informatics”[Mesh]) “INSPEC Controlled Terms”: emergency management AND “INSPEC Controlled Terms”:disaster

### Inclusion and Exclusion Criteria

**Figure 3:**
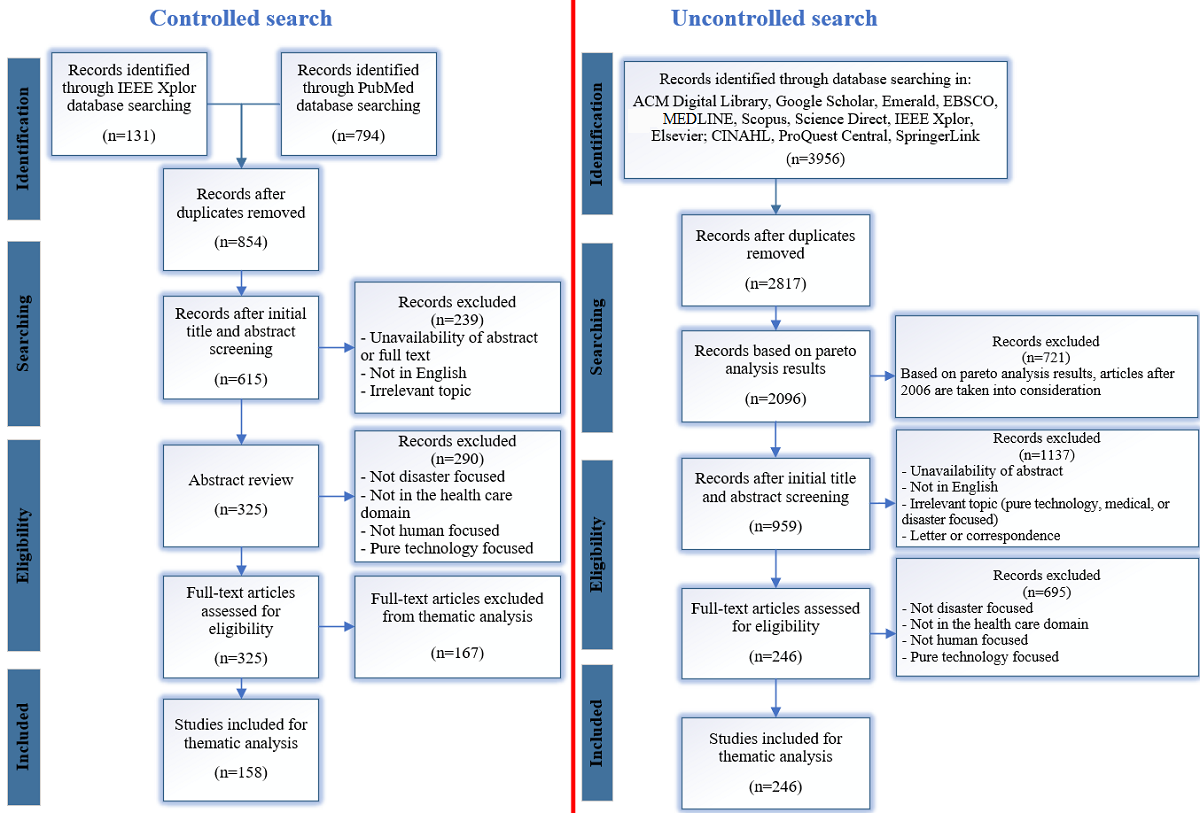
Controlled and uncontrolled searching steps. CINAHL: Cumulative Index to Nursing and Allied Health Literature; EBSCO: Elton B Stephens Company.

Both search procedures were iterative and captured relevant articles regardless of their research design, methodology, and quality (recommended by Valaitis et al [14]). The selected articles were in the English language and explicitly referred to eHealth technologies and their applications in disaster management and/or disaster medicine. The examined databases, the overall flow of study selection, and inclusion and exclusion criteria for the uncontrolled and controlled search are depicted in Figure 3.

In the uncontrolled search to facilitate the preliminary eligibility examination phase, Pareto analysis was used to exclude a larger number of articles in a shorter time without affecting the quality of the results. Pareto analysis is a well-established statistical technique in the business and management field known as 80/20 rule, that is, 20% of the major tasks and activities can generate 80% of the benefit of doing the entire job [15]. This method helped the researcher, instead of dealing with all the studies, to focus on those that had the most significant impact on the research output. Pareto analysis was performed based on the number of technology types used in DMC for health care purposes in each year, called *technology saturation*. The rationale for technology selection in Pareto analysis is rooted in the rapid technological revolution or advancement in the field of computation.

**Figure 4:**
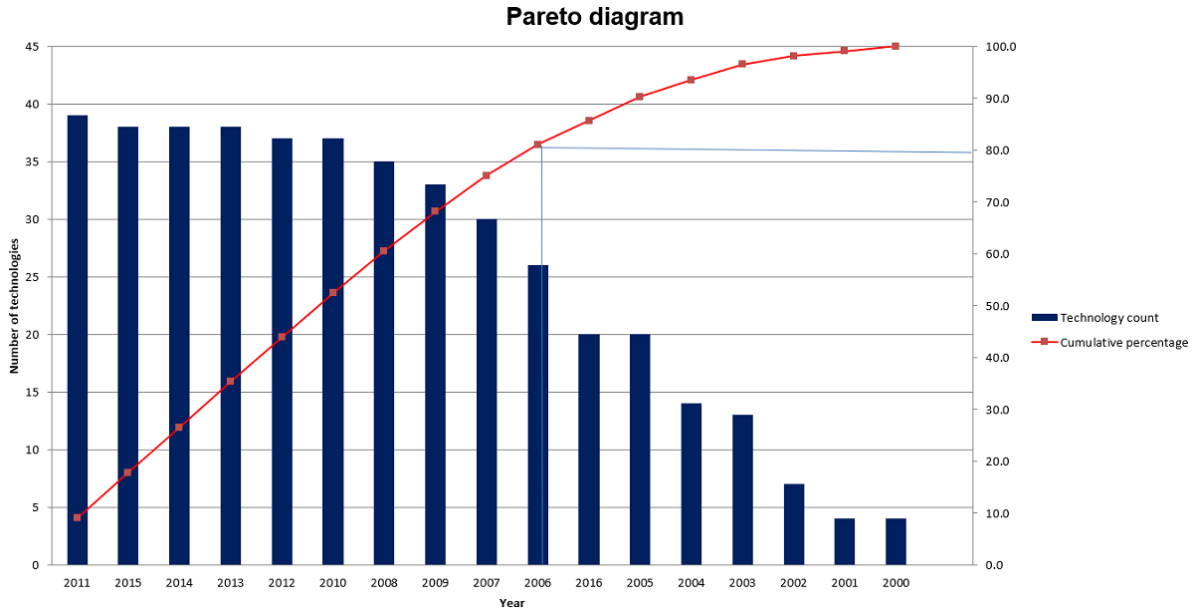
Pareto analysis diagram in uncontrolled search.

By following the Pareto formula [15,16], only articles after 2006 were included for review. The Pareto analysis diagram is depicted in Figure 4.

### Data Analysis Method

After selecting the studies for the in-depth review, in controlled and uncontrolled rounds, their full text was added to EndNote (Web of Science Group) [17] and NVivo (QSR International) [18] databases for qualitative analysis, summarizing, and drawing conclusions. Thematic analysis [19] and content analysis [20] were used to interpret different aspects of each study and identify the main themes. The analysis mainly intended to identify the important features related to this research and categorizing the findings into different thematic groups. These themes were inductively derived from the data for which we first familiarized ourselves with the data sample by reading a number of articles. Each created theme consisted of information that concentrated on or covered a particular aspect related to eHealth in disaster management or disaster medicine. The themes included potential technologies and their functions, attributes, stakeholders, and other related concepts.

On each theme, a conventional content analysis was performed to interpret the findings of the theme. The analyses identify the opinions and general trends in eHealth adoptions and applications within disaster management and disaster medicine fields. Finally, the Delphi method was conducted in 2 rounds in which the fields ‘experts evaluated the initial DEH scope. The results of this evaluation are reflected in the reported DEH scope in this paper.

## Results

### Disaster Types and Phases in DEH

Identification of disaster type is necessary to select appropriate approaches to DEH for particular cases. The research results highlighted a high diversity in the literature with regard to disaster types. A comprehensive list of disaster types was extracted based on the CRED database [2].

**Figure 5:**
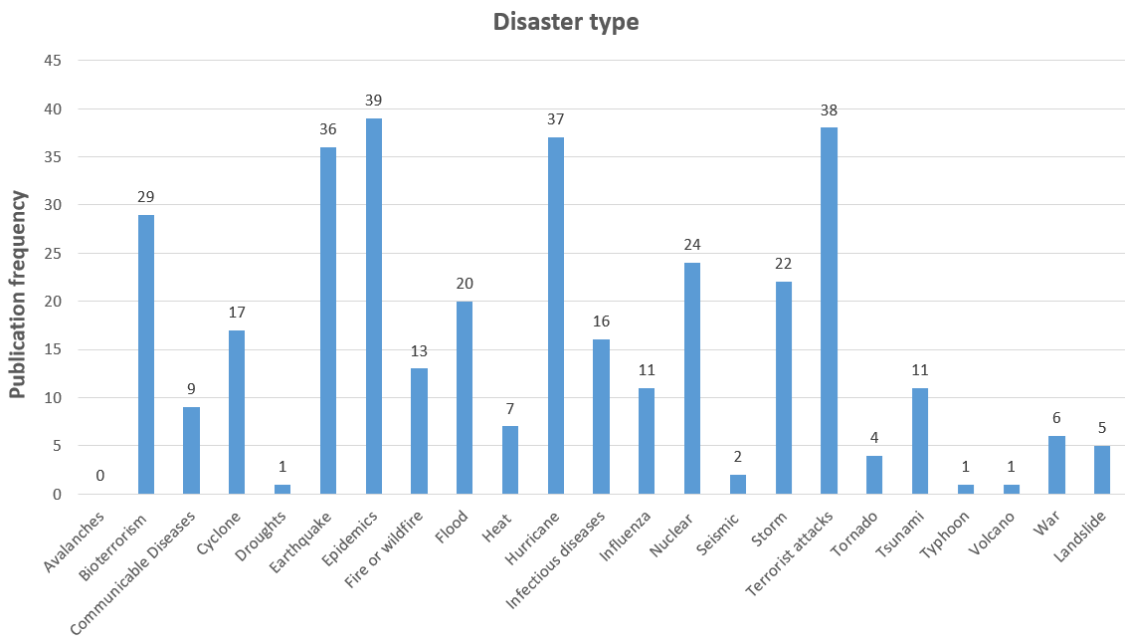
Identified disaster types in disaster eHealth scoping.

Comparison of this disaster’s classifications with the research findings reveals that DEH can cover almost all types of disasters. As in the research we searched for eHealth applications within DMC, this, in turn, may be interpreted as eHealth can be used to support disaster management and disaster medicine activities across a wide range of disaster types, regardless of their sources. The detailed findings of the identified disaster types are presented in Figure 5.

On the basis of the frequency of disaster types found in the scoping results, eHealth technologies’ usage is distributed across a broad range of disaster types. Nevertheless, the use of eHealth in epidemics (39/349, 11.1%), terrorist incidents (38/349, 10.8%), hurricanes (37/349, 10.6%), and earthquakes (36/349, 10.3%) is discussed more than in other disaster contexts in the literature. This may mean that these areas are likely to be more researched either because of researcher interest or the frequency of their occurrence.

To identify the disaster phases on which eHealth technologies can be used, within the DEH scope, it was referred to as four-phase DMC: mitigation, preparedness, response, and recovery [3]. The examination of the literature suggests that eHealth technologies can be used in all disaster phases, as according to the literature in all phases eHealth technologies were used. However, they were mostly reported as being used or useful in the preparedness (255/513, 49.7%) and response (150/513, 29.2%) phases in contrast with mitigation (49/513, 9.5%) and recovery phases (59/513, 11.5%).

### Technologies Within DEH

The scoping analysis indicates an extensive variability in the list of identified technologies from different domains, most commonly related to information system and telecommunication, but extended to areas such as artificial intelligence and robotics. To reduce this complexity, technologies were demonstrated in a hierarchical representation mapped to an existing hierarchical taxonomy of eHealth technologies. Among the consulted databases, PubMed, CINAHL (Cumulative Index to Nursing and Allied Health Literature), and EBSCO (Elton B. Stephens Company) Health databases have a taxonomy of eHealth from which PubMed was chosen because of its comprehensiveness, quality, and equality of depth and breadth of the field. In CINAHL, database subject headings for eHealth are slightly different and at a higher level. In the EBSCO Health database, only *internet* was the most suitable thesaurus term for eHealth, as the rest are mostly related to health care subjects.

**Figure 6:**
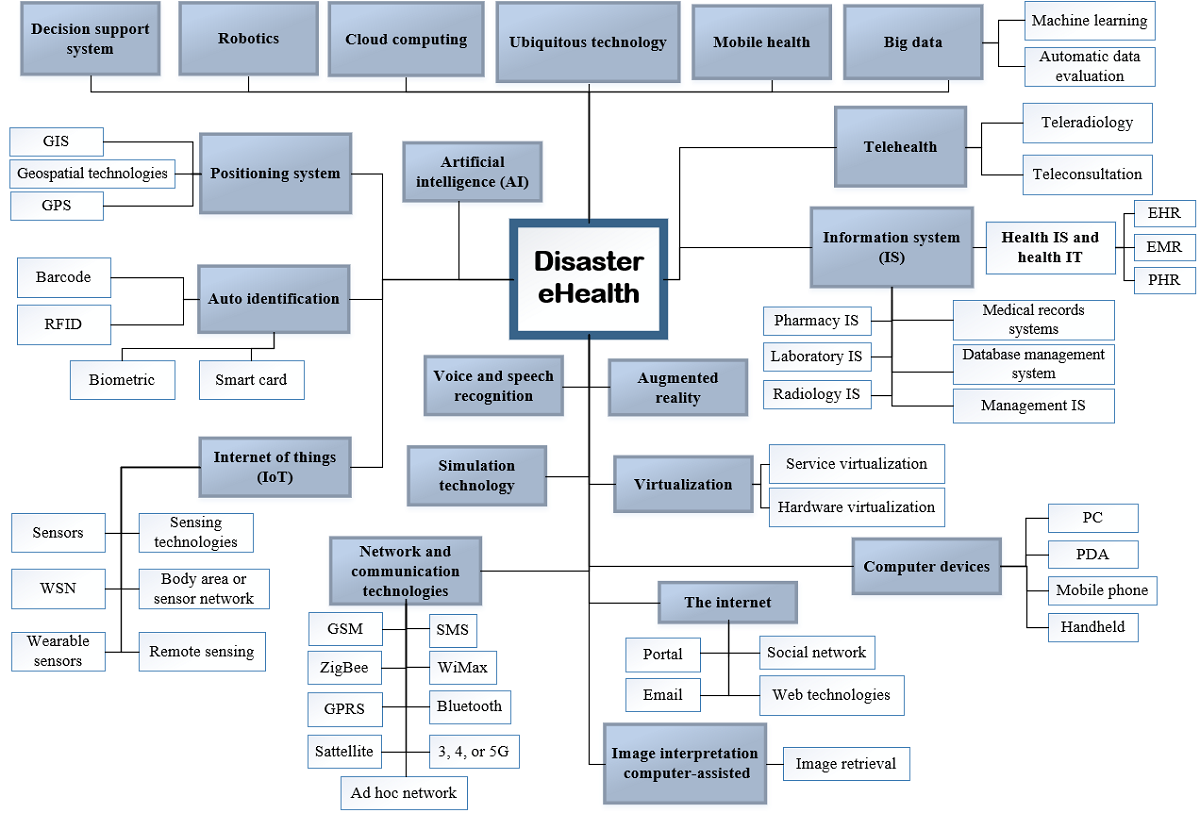
Technologies within disaster eHealth scope. EHR: electronic health record; EMR: electronic medical record; GIS: geographic information system; GPRS: General Packet Radio Service; GSM: Global System for Mobile Communications; IS: information system; IT: information technology; PDA: personal digital assistant; PHR: personal health record; RFID: radio-frequency identification.

PubMed places health care computer and digital technologies in the category of *information science* with *medical informatics* as a subcategory. On the basis of the PubMed taxonomies, the identified eHealth technologies related to DEH were mapped to these top-level classifications and then further classified into low-level technologies for a number of categories (Figure 6). In this diagram, the main technologies are shaded. This diagram may help to identify eHealth technologies that can be used in DMC to facilitate its activities.

DEH embraces a wide range of technologies to support health care activities in different disaster types and phases. A number of these technologies are specifically designed for health care environments such as *electronic health record* (EHR), *teleradiology*, and *radiology information system*. In contrast, there are some technologies that are not designed for health care environments. However, based on their positive outcomes in other areas, the health care sector has started using them for the same purposes or other clinical purposes. Technologies such as *auto identification*, *decision support system [DSS]*, or *big data* are among these technologies.

There is a vast range of eHealth technologies in the DEH scope. Among the identified eHealth technologies, our results indicate *information system* and its subtechnologies present prevailing functions and applications in DMC. Furthermore, technologies range from well-established fields, such as *DSSs*, *telehealth*, and *information system*, to the new emergent fields, such as *Internet of Things* (IoT), *augmented realities*, and *big data*.

### Technology Functions Within DEH

**Figure 7:**
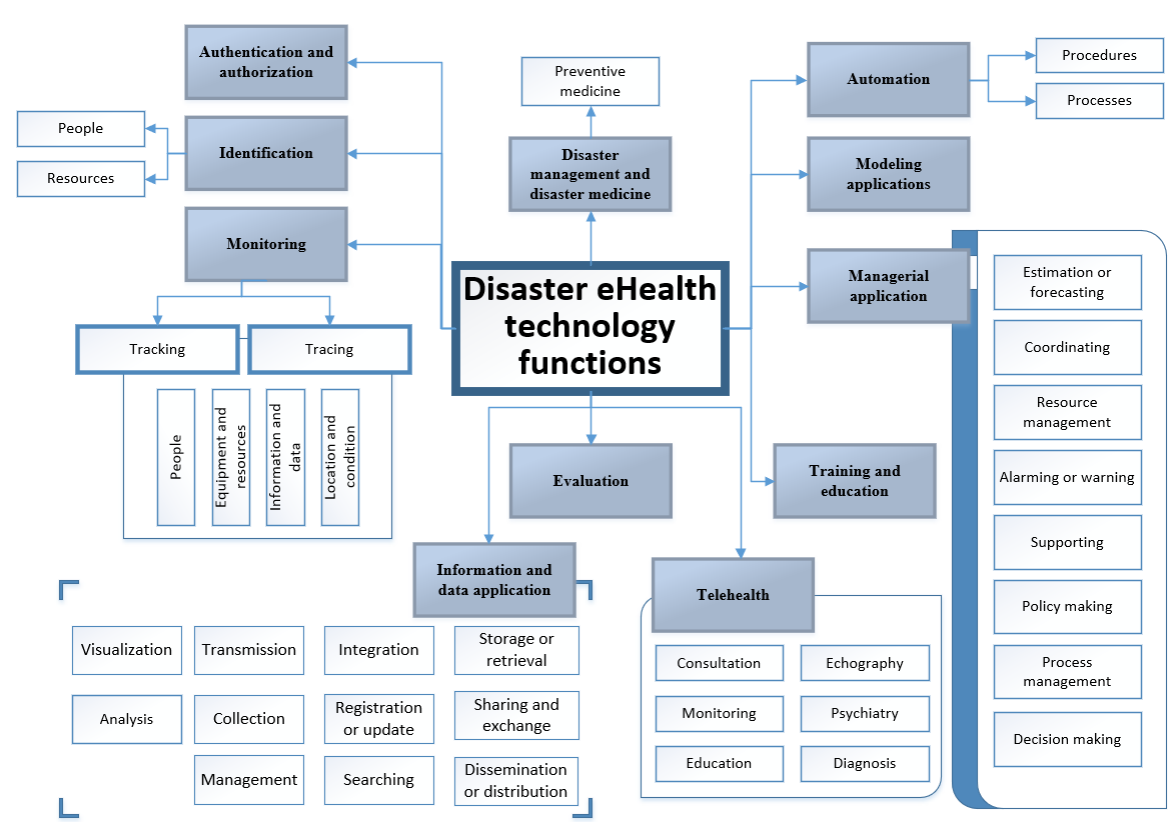
Technology functions within Disaster eHealth.

The identified eHealth technologies could serve specific functions within the DMC and DEH scope. Our thematic analysis identified these functions and highlighted the major ones and classified them based on their features and applications (Figure 7). These functions demonstrate how DEH can support disaster management and medicine people in their tasks.

### Attributes of Solutions for DEH

The identified technologies and their applications support different attributes that could be important in disaster management or disaster medicine activities. On the basis of the thematic and content analysis results, Textbox 2 presents the list of the most important attributes found for eHealth technologies in DEH. These attributes could potentially facilitate disaster preparation, response, or recovery activities.

**Attributes of eHealth technologies in Disaster eHealth.**

Feature name

- Accessibility
- Accountability
- Accuracy
- Availability
- Awareness
- Collaboration
- Completeness
- Computerization
- Confidentiality
- Consistency
- Continuity
- Control
- Cooperation
- Coordination
- Effectiveness
- Efficiency
- Immediacy
- Integration
- Interoperability
- Localization
- Optimization
- Protection
- Quality
- Readiness
- Real time
- Recognition
- Relevancy
- Reliability
- Responsibility
- Robustness
- Safety
- Scalability
- Security
- Sustainability
- Telemetry
- Usability
- Web base

### DEH Purposes

On the basis of Figures 6 and 7, the overall purposes of the identified technologies and the functions within the DEH scope can be divided into 2 main clusters: *clinical* and *nonclinical*. Nonclinical purposes can be further divided into administrative, education, training, and research. These DEH purposes can be defined as follows:

1. Clinical purposes: All the tasks and activities, the objectives of which are rooted in providing or expanding disaster health care services for the population.
2. Administrative purposes: All the tasks and activities, the objectives of which directly or indirectly facilitate providing or expanding disaster health care services for the wider population and can cover health care administrative procedures from admitting patients to discharging them or making patient information transfer possible.
3. Education and training purposes: Covers activities where the main purpose is to train and prepare citizens or special groups such as responders for different disaster phases.
4. Research purposes: The cases where their aims are directly or indirectly related to investigation and research. They intend to improve quality, cost-effectiveness, and equity of access to health care services within the DMC.

On the basis of these definitions, a number of example activities for each group of disaster phases are shown in Table 2.

**Table 2:** Disaster eHealth activities examples with regard to disaster eHealth purposes.

Purpose and disaster phase		Sample of activity
**Clinical**		
	Mitigation	Medical planning
	Preparedness	Transferring and sharing of medical information
	Response	Remote triaging of injured patients before arriving at hospitals
	Recovery	Helping injured patients to recover at home
**Administrative**		
	Mitigation	Preparing humanitarian aid program
	Preparedness	Disseminating predisaster warnings
	Response	Providing automation to help responders on documentation during disaster response
	Recovery	Identifying and locating missing children
**Education and training**		
	Mitigation	Educating people on the foundational elements of preparedness
	Preparedness	Training the practitioner on disaster skills
	Response	Providing continuous medical education for practitioners without enough experience
	Recovery	Providing tele-education supports and services
**Research**		
	Mitigation	Studying the previous disasters and research on the probability of their occurrence and their consequences
	Preparedness	Carrying research on required competencies for the response time
	Response	Not applicable
	Recovery	Conducting studies to identify the long-term impacts of disasters on people’s health

### DEH Stakeholders

**Figure 8:**
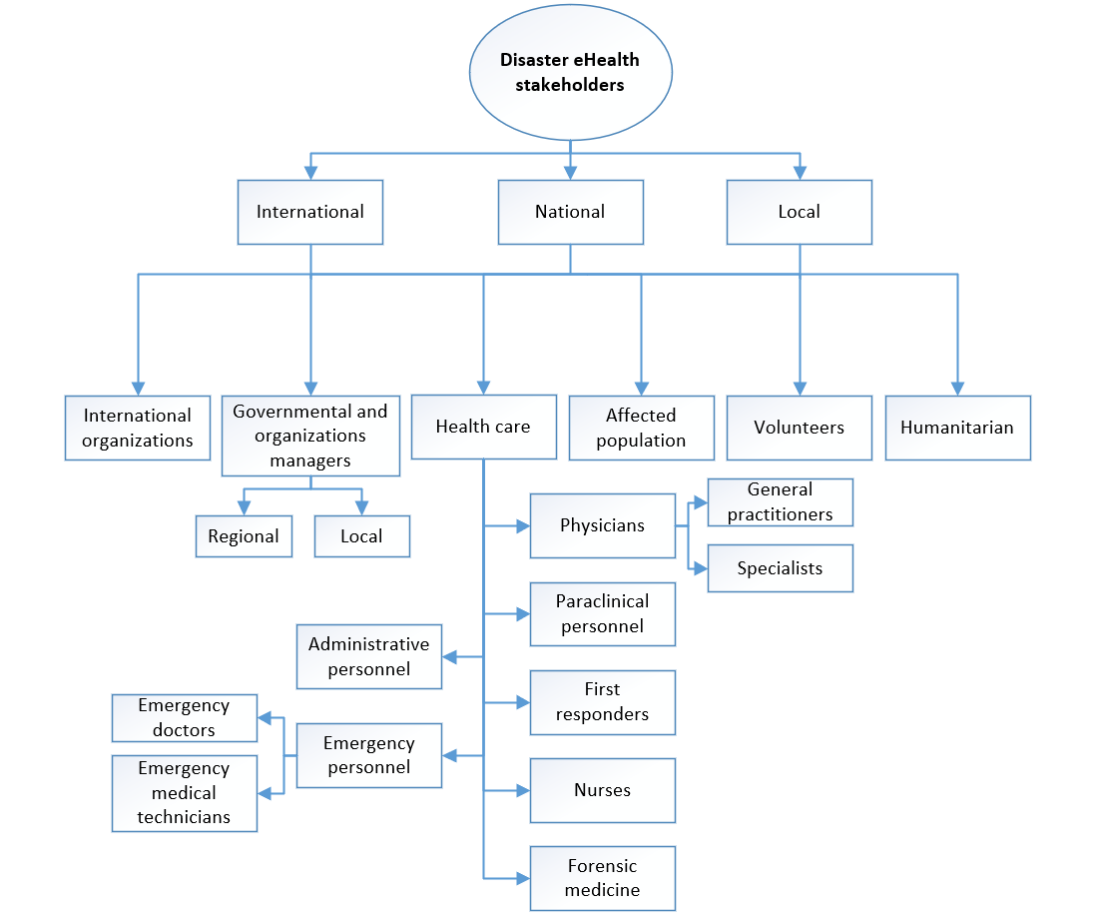
Disaster eHealth stakeholders.

The review revealed a wide range of DEH stakeholders, targets, or users of the specified technologies and functions at local, national, and international levels. The *international* level refers to the international organizations that work in any areas that directly or indirectly can have a role in any disaster phase regardless of their nature, and their branches and their supports cover almost all countries; examples of which include the World Health Organization, Red Cross, and Red Crescent. In contrast, the *national*-level organizations exist, and their rules are applied within the boundaries of each country and could vary from country to country. At the bottom of the hierarchy, *local* organizations are located in the country at the subnational level; they work under national or governmental organizations and follow their rules. These organizations are responsible for the needs and demands of specific regions or areas; they are not supposed to make any rules, are just executors of the governmental rules, and need to report to the national organizations. Police and fire bureaus and the regional health care environments are considered at this level. On the basis of their level, we can set out the categories and subcategories in terms of different parties (Figure 8).

### Services Within DEH

On the basis of the different parties and their needs in each phase of a disaster and the supported technologies and functions, DEH covers a diverse spectrum of services and purposes within the DMC. These services, from a high-level view, can be categorized into 3 levels:

1. Operational-level services: The function of this group of services is to support or control operations against rules and standards and encompass day-by-day decisions. Although diversity in these services can create islands of automation, they make operations more efficient. A large number of services in DMC and DEH can be categorized under this level, such as rapid victim identification, victim tracking, damage assessment, and critical resource distribution.
2. Tactical-level services: The purpose of this range of services is to support management and provide interconnection among different parties or organizations through diverse information management tools. These services take care of medium-term planning and are used in creating procedures. Under this level of service are categorized DSSs as well as capacity assessment databases, health information exchange, and appropriate allocation of resources.
3. Strategical-level services: The purpose of these level services is to support the system as a whole, and their output is mostly policies and overall structural decisions, either among an organization’s functions or among other organizations. For this group of services, information planning tools are used, and integrated infrastructure and global compatibility are essential. Above all, few services are available for this stage not only in DEH but also in conventional disaster management and medicine. These services cover long-term, complex, and nonroutine planning in DMC and DEH, such as planning for vulnerable population needs and safety, long-term care, or cloud-based coordination. To be useful, strategy must translate into tactics and delivery so that services defined at this level will have related examples at both the tactical and operational levels.

According to this classification and explanation, we can place these services for each disaster phase within the DEH scope. Some examples are presented in Table 3.

**Table 3:** Disaster eHealth service level examples.

Service level and disaster phase		Sample of service
**Strategic**		
	Mitigation	Enhancing patient education and empowerment Analyze out-of-hospital emergency medical services
	Preparedness	Preparing back-up communications systems Education and training of health workers
	Response	Monitor, aggregate, and analyze social media data Cloud-base coordination
	Recovery	Long-term care Injured people information
**Tactical**		
	Mitigation	Information integration Medical record sharing within and across institutions
	Preparedness	Resource database Decision support systems for bioterrorism preparedness
	Response	Dynamic information collection Coordinating the distribution of the available medical resources
	Recovery	Supply chain management Integrate the delivery of care after disasters
**Operational**		
	Mitigation	Organize medical resources Analyze daily operations in emergency departments
	Preparedness	Remote sensing Response plans that rely on local hospitals
	Response	Situational awareness Electronic triage tag
	Recovery	Psychiatrist video conferencing Evaluation and identification of psychological problems

## Discussion

### Principal Findings

eHealth can facilitate health care data exchange and dissemination, improving communication, support, and education among communities, health care professionals, and their patients [21]. These points also apply to DMC activities that can be facilitated and supported by eHealth technologies. Nevertheless, there is a paucity of studies in this area, and eHealth technologies have not been systematically integrated into disaster management nor in disaster medicine. Integrating and utilizing eHealth technologies throughout the DMC on a systematic rather than ad-hoc basis could enhance the efficiency and effectiveness of both disciplines. This integration can improve the performance of health care and enhance the quality of its delivered services before, during, and after disasters. This differentiates DEH from its constituent fields that only deal with health care in normal medicine (such as eHealth) or have a limited technology usage (such as disaster management and disaster medicine). DEH also covers the whole range of DMC activities and addresses all disaster phases; it accommodates all disaster types and is not a disaster-specific model.

In this study, the initial scope of the DEH is defined. DEH tries to maximize health care engagement in and integration into the DMC because an effective and successful response is almost unachievable without appropriate levels of different sectors’ readiness, including health care. By integrating health care into the predisaster phases, health care can be shifted from a reactive to a proactive system when disasters occur. In this regard, the variation of eHealth technologies within the DEH scope offers a broad range of functions and applications to facilitate health care management and delivery. For example, as education and communication play a vital role, telehealth and social networks could be useful in raising public awareness or providing remote and special clinical education for physicians. The importance of this application in educating the general population [22] or providing patient care and education by physicians [23] is acknowledged in recent studies to reduce the health consequences of the most recent disaster, COVID-19. In addition, by using IoT technology, tracking and sharing medical resources or data may be carried out automatically, which, in turn, could result in enhancing data accuracy.

The technologies themselves can also be integrated to optimize the outcome. For example, the collected data through IoT could be aggregated by cloud computing and then analyzed with big data analytics tools to support strategic disaster management planning or possibly scenario prediction. Such a framework for technology integration was proposed by Madanian and Parry [24] and recently been applied to prevent the spread of COVID-19, as suggested by Adly et al [25]. In addition, as assessing a variety of national and local medical information is a disaster mitigation requirement [26], it could be facilitated by using big data and cloud computing. Some of these technologies could offer integration within and across disaster phases. For example, continuous clinical monitoring of people with chronic diseases is an available service through the IoT and mobile health in normal medicine. If the service becomes common for the postdisaster phase, it could support continuity of care with minimal care plan disruption. This service on its own or by integrating with EHR may improve follow-up treatment and allow better care regardless of the geographical location of physicians and patients or providing better health care when there is a lack of specialists or experts in disaster-stricken areas. This area has attracted an exponential interest after the COVID-19 pandemic and has been recognized by different researchers as a viable solution to support health care professionals and reduce pressure on health care systems [27,28].

More recently, the IoT, big data, and cloud computing have attracted an exponential interest in automatic data gathering, integration, and analysis for data sharing and decision support applications. The usage of specific eHealth technologies, such as *EHR* and *telehealth*, are currently limited in disaster settings. However, recently, they have attracted significant interest in responding to COVID-19, but most applications are in developed countries, and most low-resource countries are still suffering [29,30]. This reflected on the broader advanced technological challenges and availability context in disaster situations. On the basis of our analysis, issues related to the availability of technology in developing countries and their security challenges and network requirements are some of the reasons that affect their usage. In contrast, the use of some technologies, such as positioning technologies and social networks, has rapidly increased because of their wide accessibility and availability.

In our research, we explicitly identified DEH stakeholders as technology users or targets who can benefit from DEH. These groups could be involved in DMC for a variety of purposes and in different positions. From a broader perspective, we can have the following groups:

- DEH seeks to enhance clinical and nonclinical personnel’s disaster-related awareness, education, elements, standards, and procedures, mainly in disaster mitigation and preparedness phases. This could possibly result in better response and in meeting wider health care demands, as health care teams are familiar with the very concept of disaster.
- For disaster management and disaster medicine people, DEH may facilitate technology adoption in their fields, one of the consequences of which, possibly, is rapid communication and data sharing among involved parties in DMC. This results in enhancing access to precise information in a timely manner, which, in turn, may lead to improving the quality of decisions while decreasing the decision-making time.
- DEH may also appeal to the general population who may be affected by different types of disasters. DEH, based on its defined goal, raises disaster awareness among all people, especially communities in disaster-prone areas. Therefore, population empowerment can be enhanced, and the population can access and use information, become familiar with disaster consequences, and be prepared for them. This will increase preparedness against disasters and if any disasters strike their areas, they are able to take care of their basic health care requirements until disaster responders arrive. Then, as disaster responders have proper training and are equipped with different types of eHealth technologies, they are able to transfer timely and accurate information from disaster sites to top authorities so that they can make appropriate decisions. This, in turn, provides better health care services to disaster casualties.

### Conclusions

Preparedness and planning to reduce the harmful effects of disasters is becoming one of the highest priorities of governments. These activities are features of disaster management and disaster medicine; disciplines that despite their long standing still generate many debates about their effectiveness and capabilities in responding properly to health care demands in major disasters [31].

This research, along with other studies such as by Sieben et al [32] and Norris et al [6], is seeking ways to develop systematic principles for coordinating disaster management and disaster medicine more effectively by incorporating eHealth technologies. These technologies may improve the response to health care demands before, during, and after disasters. They can be used to improve overall disaster management, facilitate response when disasters occur, enhance support after disasters, and keep records for better future preparedness. Although some eHealth tools are employed in disaster settings [31], their applications are not generally systematic and routine; rather, they are more ad hoc. The most recent example is the COVID-19 pandemic that has increased the interest in eHealth technology adaption to have a more effective response while reducing the strain on health care. However, the issues with the utilization of these technologies are still their ad hoc usage and lack of system integration with health care mainstream activities. Moreover, there are different examples of using eHealth technologies to respond and recover from COVID-19 but with no overarching guidelines of when and how to apply them, and in some cases, training is required to promote success.

The DEH domain has been introduced mainly to facilitate addressing the current challenges within disaster management and disaster medicine that hinder their operations and created many debates regarding their efficiency and effectiveness. DEH emergence contributes to the design of a systematic model for eHealth technologies that are currently used in nondisaster circumstances but have the potential to be used in disaster situations along with those technologies that were previously used in DMC and had a significant impact on DMC operations.

DEH takes advantage of eHealth technologies to facilitate DMC tasks and activities, enhance their efficiency and effectiveness, and improve health care delivery to a wider population regardless of disaster types and phases.

In this research, we extensively reviewed the academic literature to define the DEH scope. We have built our scope mostly based on available international hierarchies to make easier embedding DEH into disaster management, disaster medicine, and eHealth fields. Some of the international hierarchies that we referred to are the disaster types offered by CRED and PubMed Medical Informatics taxonomies. However, this work is mostly limited to academic and scientific publications, and gray literature is not extensively reviewed.

eHealth technologies are developing rapidly, and the COVID-19 pandemic has revealed some of the eHealth potentials in practice on addressing health care issues. Therefore, we would consider and continue to work on the DEH model and add the most recent applications, such as contact tracing, into the model in the near future.

## Abbreviations

CINAHL: Cumulative Index to Nursing and Allied Health Literature
CRED: Centre for Research on the Epidemiology of Disasters
DEH: disaster eHealth
DMC: disaster management cycle
DSS: decision support system
EBSCO: Elton B Stephens Company
EHR: electronic health record
IoT: Internet of Things
